# Exploring Patient Needs and Preferences in CKD Education: A Cross-Sectional Survey Study

**DOI:** 10.34067/KID.0000000000000369

**Published:** 2024-01-25

**Authors:** Rebecca J. Allen, Alex Nakonechnyi, TramAnh Phan, Catherine Moore, Erika Drury, Rickinder Grewal, Scott E. Liebman, David Levy, Fahad Saeed

**Affiliations:** 1 Center for IT Engagement (cITe), Mount St. Joseph University, Cincinnati, Ohio; 2 Division of Nephrology, Department of Medicine, University of Rochester Medical Center, Rochester, New York; 3 Division of Nephrology, Division of Palliative Care, Departments of Medicine and Public Health, University of Rochester School of Medicine and Dentistry, Rochester, New York

**Keywords:** CKD, dialysis, patient satisfaction, patient-centered care, progression of renal failure, quality of life, social health justice

## Abstract

**Key Points:**

- This largest to date patient survey study explores what patients with kidney disease want to know about treatments, such as dialysis or conservative management.
- A surprising number of patients want extensive doctor-like education, but are willing to spend only several hours on education.
- Patients are notably open to online and digital educational modalities—technology may allow for individualized and ongoing patient education.

**Background:**

Despite efforts to educate individuals with CKD and thereby improve outcomes, studies have shown that a significant number of patients still report poor CKD knowledge. Thus, understanding patient needs and preferences is crucial for the development and implementation of an effective CKD educational program.

**Methods:**

A paper survey was distributed to patients with CKD 21 years and older at a tertiary care hospital's outpatient nephrology clinic in Rochester, NY. Data on patient demographics; print and technological literacies; and preferences regarding topics, instructors, class formats, session frequency, duration, and peer support were gathered.

**Results:**

The mean age of 337 patients was 65 years (±12.33 years), and the self-identified races were American Indian or Alaska Native (<1%), Asian (3%), Black (12.17%), Native Hawaiian or other Pacific Islander (<1%), White (83%), and Other (2%). Most of the patients (69%) never needed help with health instructions, and 68% of patients used a smartphone or computer every day. Key topics identified by patients included the definitions of CKD, creatinine, and GFR and information on kidney diet. Seventy-three percent of patients desired more than basic CKD information, with one in five even wanting to know everything a doctor knows. Forty-six percent were willing to attend classes, and 33% preferred using digital (video, computer, or smartphone) modalities. Patients were willing to attend an average of 3.6 classes, and most preferred hour-long classes. Most of the patients (46%) preferred a doctor as the educator, and 53% expressed interest in connecting with fellow patients for peer support.

**Conclusions:**

Most patients with CKD are interested in comprehensive education about their disease. This research may offer insights into the optimal content and delivery of CKD educational programs by elaborating on patients' needs and the integration of online modalities to deliver content. Future person-centered educational programs for people with CKD are needed.

## Visual Abstract

**Figure:**
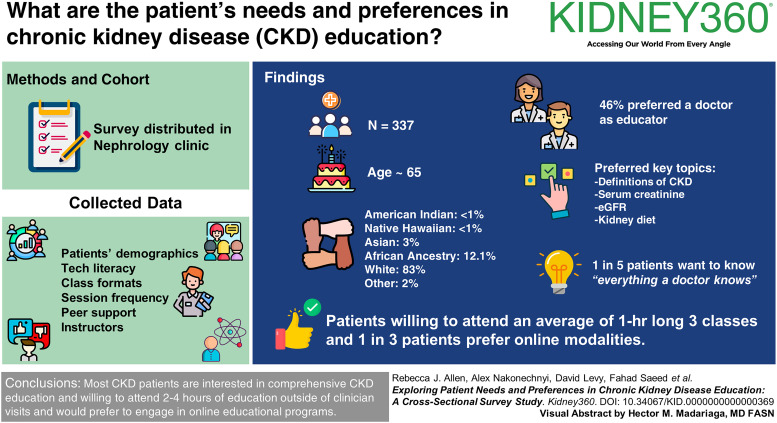

CKD affects an estimated 15% of the US population.^1^ Despite this high prevalence, many individuals remain asymptomatic for extended durations, and a CKD diagnosis often comes as a distressing surprise.^2,3^ Numerous studies have demonstrated the positive effect of CKD patient education, including increased engagement in kidney therapy decision making, reduced mortality, delayed CKD progression, and early access creation for those who are likely to progress to ESKD.^4–7^ Yet, CKD education is ideally individualized and thus challenging because varying educational levels, socioeconomic disparities, and racial disparities are common in the CKD patient populations,^8–10^ and individually, each patient brings their own needs and contexts.^11,12^ These difficulties and complexities are often compounded by the cognitive and functional decline associated with disease progression^13,14^ and the high symptom burden of advanced CKD.^15^

In light of these considerations, our group, comprising academic researchers, nephrologists, a kidney palliative care physician, and a health literacy specialist, conducted a needs analysis among people with CKD. To aid in the development of an educational program, we surveyed patients to assess their preferences for CKD-related knowledge, information quantity, educational modality (format), session duration and frequency, information sources, instructor preference, and option of peer support.

## Methods

### Setting, Study Population, and Data Collection

We conducted this study at Strong Memorial Hospital's outpatient nephrology clinic in Rochester, NY, as a quality improvement project exempt from IRB approval. Eligible participants included patients 21 years and older who arrived to see a nephrologist. Non-English speakers were excluded. Paper surveys were provided to patients at the clinic reception. Patients completed surveys either independently or with assistance from caregivers in the waiting area. Completed surveys were collected by clinic staff and stored in a locked cabinet. Staff members were available to clarify survey questions and assist patients with visual impairments. A research coordinator entered the deidentified data into an Excel spreadsheet in a password-protected computer, and the paper surveys were subsequently shredded.

### Measures

Survey items were created on the basis of prior literature,^16,17^ several rounds of discussions among the authors, clinical experience, and the research objectives. After eight rounds of face validity and readability testing, a pilot test involving 50 patients provided additional feedback. After testing, we made minor adjustments, such as enlarging font size, optimizing layout clarity, and rectifying minor language errors. Pilot test responses are included in the results. The following domains were included in the survey.

#### Sociodemographic Variables

Data on age, self-identified sex, self-identified race, marital status, educational level, duration of CKD diagnosis, and number of prior nephrologist visits were collected (Table 1).

**Table 1:** Demographic information of survey respondents

Age, yr	Mean age (SD) (range), 65±12.33 (18–94)

Participant Characteristics	Count	Relative Percentage
**Sex**		
Male	143	42.35
Female	192	56.97
Other	2	0.59
**Hispanic or Latino?**		
Yes	9	2.67
No	325	96.44
**Race**		
American Indian or Alaska Native	3	0.89
Asian	11	3.26
Black	41	12.17
Native Hawaiian or other Pacific Islander	2	0.59
White	281	83.38
Other	8	2.37
**Marital status**		
Married	190	56.38
Divorced	41	12.17
Widowed	30	8.90
Separated	8	2.37
Single (never married)	50	14.84
Committed relationship, not married	16	4.75
**Educational attainment**		
Less than high school	16	4.75
Grade 12 or GED	67	19.88
Associates or some college	108	32.05
Bachelor's degree	72	21.36
Graduate or professional degree	69	20.47
**How long have you known about your CKD?**		
I just learned I need a kidney doctor	33	9.35
Less than 3 mo	10	2.83
Less than a year	29	8.22
1–3 yr	107	30.31
More than 3 yr	139	39.38
No answer	18	5.10
Other (open-ended)	17	4.82
**How many times have you seen your kidney doctor?**		
One time	54	15.30
Two times	28	7.93
Three times	39	11.05
More than three times	188	53.26
No answer	25	7.08
Other	19	5.38

Note: In instances of race and sex, participants could select multiple options.

#### Health and Technological Literacy

Questions targeted possible assistance required for reading instructions from the doctor or pharmacy, frequency of internet and mobile device use, as well as use of social media (Table 2).

**Table 2:** Self-reported patient health and technological literacy

Health Literacy Aspects	Count	Relative Percentage
**Need assistance reading instruction from doctor or pharmacy**		
Never	244	69.12
Rarely	46	13.03
Sometimes	30	8.50
Often	10	2.83
Always	1	0.28
Never	0	0.00
No answer	5	1.42
Other (open-ended)	17	4.82
**Frequently (at least daily) use the internet and technologies such as cell phone, tablet**, ***etc***.		
Yes	239	67.71
No	42	11.90
Not sure	6	1.70
No answer	43	12.18
Other (open-ended)	23	6.52
**I use social media sites (*e.g*., Twitter, Facebook, Instagram, Snapchat)**		
Daily	117	33.14
A few times per week	25	7.08
A few times per month	11	3.12
Rarely	30	8.50
Never	105	29.75
Not sure	4	1.13
No answer	39	11.05
Other (open-ended)	22	6.23

#### Preferred Educational Topics

Participants chose and ranked 3–5 potential topics for CKD and related education (Table 2).

#### Desired Amount of Information

Participants were asked how much information about CKD they wanted—basic necessary information, basic necessary information plus some more, a lot of information, or everything a doctor knows (Table 3).

**Table 3:** Topics most important to patients

Possible Educational Topics	Count	Weighed Score	Scaled 1–5
Definition of CKD	154	596	5.00
Definition of creatinine and GFR?	175	596	5.00
Kidney diet	115	545	4.57
Ways to slow the progression of kidney disease	122	480	4.03
Meaning of having protein in urine	52	351	2.94
Meaning of blood and urine tests	97	331	2.78
Ways to self-manage kidney disease	71	228	1.91
Dialysis options	85	207	1.74
How much water to drink	60	163	1.37
How to avoid dialysis	52	150	1.26
Ways to improve quality of life	39	99	0.83
End-of-life planning	24	54	0.45
Life expectancy	10	29	0.24
Coping with kidney disease	7	17	0.14
Access to social programs for people with kidney disease	7	13	0.11

#### Preferred Modalities of Education

Participants were asked if they would like to attend classes (yes, no, or other as an open-ended response) and what the potential obstacles (work schedule, transportation, already know enough, lack of time, cost, family commitments, and other as an open-ended response) to attending classes may be. Participants chose how they learn best—lectures, classes with other patients with kidney disease, smartphone apps, videos, computer, and pamphlets/brochures” (Table 3).

#### Preferred Frequency and Duration of Classes

The survey asked the minimum and maximum number of classes patients would be willing to attend; the survey also asked patients about the length of classes (Table 4).

**Table 4:** Amount of CKD knowledge, class format, duration, frequency, instructor preferences, and peer support

Patient Preferences	Count	Relative Percentage
**How much information desired**		
Basic necessary	24	6.80
Basic necessary plus some more	93	26.35
A lot of information	90	25.50
Everything a doctor knows	76	21.53
No answer	42	11.90
Other	28	7.93
**Willingness to attend class**		
Yes	161	45.61
No	153	43.34
Other	19	5.38
No response	18	5.10
**Obstacles to class attendance**		
Work schedule conflict	30	8.50
Transportation	23	6.52
Already know enough	31	8.78
Lack of time	15	4.25
Cost (if not covered by insurance)	8	2.27
Family commitments	7	1.98
No answer	146	41.36
Other	93	26.35
**Ways one learns best**		
Lectures	83	23.51
Interactive classes	55	15.58
Smart phone apps	25	7.08
Videos	40	11.33
Computer	53	15.01
Pamphlets/Brochures	34	9.63
No answer	47	13.31
Other	16	4.53
**Minimum number of classes willing to attend**		
1	84	23.80
2	67	18.98
3	68	19.26
4	14	3.97
5	3	0.85
6	14	3.97
No answer	82	23.23
Other	21	5.95
**Maximum number of classes willing to attend**		
1	40	11.33
2	33	9.35
3	57	16.15
4	42	11.90
5	25	7.08
6	52	14.73
No answer	89	25.21
Other	15	4.25
**Desired length of classes, h**		
1	191	54.11
2	62	17.56
3	1	0.28
No answer	27	7.65
Other	20	5.67
**Desire to attend together with caregiver**		
Yes	104	29.46
No	170	48.16
No answer	60	17.00
Other	19	5.38
**Desired instructor for class**		
Kidney doctor	163	46.18
A trained nurse	48	13.60
A trained patient with kidney disease	10	2.83
No answer	43	12.18
Other	22	6.23
**Should there be an option to connect with other patients with kidney disease to create a peer support system?**		
Yes	188	53.26
No	66	18.70
No answer	65	18.41
Other	34	9.63

#### Support from Caregivers and Educational Providers and Connectivity with Peers

Patients were asked whether they wanted caregivers to attend. Patients were also asked, “Who should be teaching the educational classes?” The options included a kidney doctor, a trained nurse, a trained patient with kidney disease, or other. In addition, patients were asked whether they would like the option to connect with peer support groups (Table 4).

### Data Analysis

R version 4.2.2 facilitated statistical analysis. Descriptive statistics, including frequency and percentage, were used. To determine patients' most important topics, we tallied the number of times each topic was selected. For these topic questions, we also assigned weights on the basis of the rank (1–5) patients assigned to each topic and scaled them on a 0–5 scale.

## Results

Table 1 presents the characteristics of the survey participants. The response rate could not be tracked because the survey was given to patients by the clinic staff who had multiple other responsibilities. A total of 337 patients completed the survey, with an average age of 65 years (SD ±12.33). 57.0% self-identified as female, 42.43% identified as male, and a minority (<1%) of respondents self-identified as another gender. For self-reported race, 2.67% identified as Hispanic or Latino. The study's participant self-identified racial distribution comprised American Indian or Alaska Native (3, 0.9%), Asian (11, 3.3%), Black (41, 12.2%), Native Hawaiian or other Pacific Islander (2, 0.6%), White (281, 83.3%), and Other (8, 2.4%). Slightly more than half (56.4%) of respondents were married. The remaining participants were categorized as divorced (12.2%), widowed (8.9%), separated (2.3%), single–never married (14.84%), or in a committed but not married relationship (4.8%). Regarding education, 4.8% had completed less than high school education, 19.9% had high school education, 32.1% had an associate's degree or some college education, 21.4% held a bachelor's degree, and 20.4% possessed a graduate or professional degree.

Table 1 also presents information on the duration of CKD diagnosis and frequency of patients' visits to their nephrologists. Most of the respondents (69.9%) had been aware of their kidney disease for over a year. When it came to medical visits, most of them (53.3%) reported having seen their nephrologist three or more times.

Table 2 presents self-reported health and technological literacy among the participants. A majority (69.1%) of the patients indicated that they never required assistance with health-related instructions from their doctor or pharmacist. A smaller portion (13.0%) stated they rarely needed such support while 8.5% reported needing assistance sometimes. For technological proficiency, the survey inquired about participants' comfort with using different forms of technology for educational purposes. As a gauge of this comfort, respondents were asked whether they used a computer or smartphone on a daily basis: 67.7% of the participants reported using either of these devices every day, and approximately one-third of patients (33.1%) checked social media accounts daily.

### Topics of Interest

Patients ranked the CKD topics of most importance to them (Table 3). The most important topics, ranking 4–5 on a five-point scale, were the definition of CKD, definitions of creatinine and GFR, information on kidney diet, and how to slow progression of CKD. Of medium importance (2–3 points) were the meaning of having protein in urine and the meaning of blood and urine tests. Less popular (<2) were ways to self-manage kidney disease, dialysis options, how much water to drink, and how to avoid dialysis. Open-ended responses (data not shown in tables) offered by patients suggested patient confusion about their kidney disease, treatments, and symptoms. For instance, patients wanted to know—how did this (CKD) happen, about potential treatments beyond transplant or dialysis, and about symptoms like itching and exhaustion.

### Extent of Education

Patients desired more than just basic information about CKD (Table 4). Only 6.8% wanted just basic information; 26.4% wanted to learn more than basic, necessary information; 25.5% wanted a lot of information; and notably, 21.5% of patients wanted to learn everything a doctor knows.

### Modality of Education

In Table 4, patients were essentially split on whether they would want to attend formal classes—45.6% would want to attend and 43.3% would not. The closed response obstacles to attending classes were work schedule (8.5%), transportation (6.5%), perception of already having sufficient knowledge (8.8%), time constraints (4.3%), possible cost if not covered by insurance (2.3%), and family commitments (2.0%). Patients provided free text comments (data not shown in tables) on this question as well, and a nuance that emerged was that patients did not necessarily lack transportation altogether, but rather had difficulties driving in certain circumstances—patients mentioned travel at night is difficult and the location may be too far.

Patients were asked to pick between traditional delivery methods (either lecture style classes or more interactive style classes) or content delivered using technology (through smartphone apps, videos, or the computer): 39.1% preferred the traditional classes (the sum of 23.5% preferring a more lecture style and 15.6% wanting interactivity) and 33.4% preferred using technology (the sum of 7.1% smartphone apps, 11.3% videos, and 15.0% computer), with the remaining minority preferring paper pamphlets and brochures (9.6%) or a different option.

### Number and Length of Classes

Table 4 presents the preferred number of classes patients are willing to attend. Approximately one-fifth of patients were open to attending five or six classes (five classes: 7.1%, six classes: 14.7%). On the other hand, 11.3% of patients would prefer a single class, 9.4% would opt for two classes, 16.2% for three, and 11.9% for four. Despite differences in attendance preferences, the majority (54.1%) preferred shorter classes of 1 hour while 17.6% preferred 2-hour classes, with <1% favoring 3-hour sessions (with the remaining patients indicating a preference for the option of other durations).

### Presence of Caregivers

Table 4 presents preferred support and education sources. Most of the patients did not want a caregiver to attend classes with them—only 29.4% of the patients answered yes, they wanted to come with a caregiver.

### Preferred Educator and Peer Support

46.1% desired education from a nephrologist while 13.6% preferred a nurse. 2.8% wanted education from another trained patient, and 19.0% preferred the other option. Among the other category, most desired a team approach involving a doctor, nurse, or dietitian. Patient–peer connections were valued for support, with 53.3% expressing interest in programs to connect them with peers.

## Discussion

This study explored the education preferences of individuals with CKD. Findings highlighted interest in CKD basics including definitions of CKD, creatinine, and GFR and information on kidney diet. Patients generally preferred in-depth information and desired 2–4 educational sessions lasting about an hour each. Most preferred not to attend classes with caregivers. Patients desired receiving education from nephrologists and valued peer connections for support. These insights can guide tailored CKD educational programs by aligning them with patient preferences.

We wish to discuss three main findings. First, this study provides important insight into what patients want to know—their high and low educational priorities. Clearly, patients wanted to seek knowledge about the basic concepts of CKD, such as knowing the definitions of CKD, creatinine, and GFR. Notably, in our study, patients also wanted to learn about kidney diet. This desire to seek conceptual understanding aligns with past qualitative research showing patients do in fact want information about their disease.^18^ Our reported patients' informational goals are in line with Kidney Disease Improving Global Outcomes guidelines^19(p. 14)^ and likely provide patients with a sense of agency (*e*.*g*., eating healthfully to slow down CKD progression).^20^ The increasingly clear patient desire to learn more about the basic concepts of CKD is understandable in the context of long-standing low awareness of CKD among the public.^21,22^ Nearly 90% of people with early CKD and close to 50% people with advanced CKD do not have awareness of their CKD diagnosis,^1^ and even with multiple visits with a nephrologist, patient understanding of CKD can remain poor.^1^ These data highlight a documented gap—a recent mixed-method study has identified the lack of information on diet as a limitation of CKD education programs.^20^ Regarding topics of lower priorities, what patients were not as interested in knowing about was kidney therapy options and end-of-life planning. On the surface, one possible explanation is that patients may have fears of mortality and disruptions to daily life,^23,24^ but another possible factor is that, despite the high mortality rates particularly among older patients with CKD,^8^ patients frequently do not fully understand that CKD may affect their life expectancies,^25^ although we did not ask patients to self-identify their current CKD stage.

Second, we note how much patients wanted to know—our study suggests that patients often wanted to obtain expansive information about CKD. Specifically, our study showed 25.5% of patients wanted a lot of information and 21.5% wanted to learn everything a doctor knows. The study also showed a majority of patients would want to attend 2–4 classes, and patients overwhelmingly wanted the classes to be an hour long. Altogether, this likely means patients on average would like to receive 2–4 hours of education outside of clinician visits. Certainly, 2–4 hours is not enough time to learn everything a doctor knows. This noticeable gap between patients' desired learning outcomes and expectations related to time investment in reaching these outcomes may be problematic. Adult learning theory suggests when adults do not reach their expected educational goals, they may respond by disengaging—that is, patients may demonstrate poor uptake of the material or may cease attending altogether.^26^ Setting learners' expectations up front—dialoging about what is achievable—is critical to successful learning.^26,27^ In other words, giving patients a clear notion of what will and will not be covered in a particular class or perhaps offering a selection of courses by subtopics (*i*.*e*., basics of CKD, diet, and so on) and providing links to additional educational materials may all be practical ways to maximize educational success by helping match patient–learner expectations with probable learning outcomes.

Third, patients were open to engaging in online educational programs. Approximately one in three participants preferred to receive their education through a technological modality, and 67.7% of participants reported using a smartphone and/or computer daily. This finding of technology use and acceptance in patients with CKD is in line with the growing adoption of technology among older adults^28^ and the rise in older adults' willingness to use digital health information sources.^29^ The gap between technology use in older and younger population is narrowing and projected to continue to narrow.^28^ Thus, patient education for older adults using technology cannot be dismissed, particularly as an area of growing utility.^30^ Moreover, technology offers some benefits that traditional education does not: One study of a CKD education app reported patients felt the app was particularly beneficial for lifestyle changes because important information could be consulted repeatedly and on demand.^31^ More research on technology and education on CKD is needed.

Our study has several strengths and limitations. Prior work with people with CKD highlighted lack of patient voices in disease self-management interventions,^32^ but ours is the largest study, to our knowledge, capturing patient views about different aspects of a kidney disease education program, although this is a single-center study. Many kidney disease education programs are developed without patient input. Our survey inquired about patient interest in what would they like to learn. The response rate of the survey could not be tracked because of minimal personnel in the clinic partly during the pandemic. In this convenience sample, we had representation of Black patients consistent with general demographics, but likely an underrepresentation for the kidney disease population^33^; we also did not offer the survey in languages other than English. The survey respondents generally had high educational attainment, so findings may be less generalizable to lower educational attainment levels. We also note that our survey participants may be at various stages of CKD, which we did not capture; our general goal was to determine on average what type of educational programming should be offered at the clinic to patients in aggregate. We also did not ask specifically whether patients would be interested in receiving education online synchronously—through platforms, such as Zoom—or asynchronously—through self-paced learning—rather we determined more general acceptance of smartphones and computers as a way to learn.

Our study has several implications. Our data suggest patients wish to know the basics of kidney disease—common terms and concepts discussed during a nephrology visit (creatinine, GFR, *etc*.) and also aspects of self-management (diet possibly as a way to slow down the progression of CKD, *etc*.). A Cochrane review has suggested association of a combined CKD education and self-management approach in improving CKD knowledge and enhancing self-efficacy, augmenting the physical domain of quality of life and possibly decreasing mortality.^34^ Web-based CKD management programs have been codeveloped with patients and caregivers and are helpful examples for implementation in clinical care.^35,36^ Given that less than one-third of survey respondents expressed a preference for their caregivers to attend classes with them and considering the substantial influence caregivers may have on medical decision making,^37^ as well as the effect of these treatment decisions on caregivers themselves, it is essential to explore the most effective way to offer caregiver education. This may include providing separate education resources for caregivers, as an optional approach. Further investigation in this regard is needed. In addition, patients have voiced that they want nephrologists' engagement in these education programs, yet prior work has shown little presence of nephrologists in formal CKD education programs and their unfamiliarity with the content of such programs.^38,39^ Thus, our study calls for nephrologists' training in patient education, group facilitation techniques, and appropriate communication skills to provide rigorous CKD education.^40^ It is also noteworthy that our study calls for development of CKD education programs run by a multidisciplinary team, including nephrologists, dietitians, and other peers with CKD.^20,41^ In particular, to further verify and implement our findings, more participatory methods that involve patients and caregivers throughout research and interventions are needed.^42^

In summary, our study sheds light on patient preferences for a CKD education program in terms of topics, depth, modality, frequency, and type of instructor. Moving forward, designing and pilot testing a person-centered CKD education program that offers topics most important to patients is multimodal (*i*.*e*., has online and in-person offerings), uses a multidisciplinary team, and offers a peer support component if needed.

## Supplementary Material

## Data Availability

All data are included in the manuscript and/or supporting information.

## Supplemental Material

Patient Survey
