# Supplementary material for: Exploring Patient Needs and Preferences in CKD Education: A Cross-Sectional Survey Study
Source: Kidney360. 2024 Jan 25;5(3):344–51. doi: 10.34067/KID.0000000000000369 (PMC11000739; doi:10.34067/KID.0000000000000369)
Supplement: Supplementary file 1 [file kidney360-5-344-s001.pdf]

Patient Survey

The kidney team at the University of Rochester is hoping to develop a program to educate patients about chronic kidney disease. We believe that we need to include patient voices in the kidney education program. We request that you take approximately 5-7 minutes to fill out this survey. Please return the survey to the front desk upon completion or give it to the technician who checked you in.

1. **What is your current age? ________**
2. **Which gender do you identify with?** (Female, Male, Other)
3. **Do you consider yourself to be of Hispanic or Latino origin?** (No, Yes)
4. **Which group or groups below best represent(s) your race?** (White, Black or African-American, Asian, Native Hawaiian or other Pacific Islander, American Indian or Alaska Native, Other: __________)
5. **What is your marital status?** (Married, Divorced, Widowed, Separated, Single, never married, In a committed relationship but not legally married)
6. **What is the highest grade or level of education you have completed?** (Grade 8 or less, Grade 9 through 11, Grade 12 or GED, College 1-3 years, College 4 years, Graduate degree)
7. **How long have you known that you have chronic kidney disease?** (I just learned that I need to see a kidney doctor, Less than 3 months, Less than 1 year, 1-3 years, More than 3 years)
8. **How many times have you seen your kidney doctor?** (Once, Twice, Three times, More than three times)
9. **How often do you need to have someone help you when you read instructions, pamphlets, or other written material from your doctor or pharmacy?** (Never, Rarely, Sometimes, Often, Always)
10. **I frequently (at least daily) use the Internet and technologies such as cell phones, tablets, etc.** (Yes, No, Not Sure)
11. **I use social media sites (e.g., Twitter, Facebook, Instagram, Snapchat)** (Daily, 1-3 times per week, 1-3 times per month, Rarely (< 1 time per month), Never, Not Sure)
12. **Please rank the top 3-5 topics that you think are most important to learn for patients with kidney disease.** (What is chronic kidney disease, What do my kidney's blood and urine test results mean?, What are creatinine and GFR?, What does having protein in my urine mean?, Kidney diet, Ways to slow the progression of kidney disease, Ways to self-manage kidney disease, How much water to drink?, End of life planning, Coping with kidney disease, Ways to improve quality of life, Dialysis options, How to avoid dialysis?, Life expectancy, Access to social programs for people with kidney disease)
13. **Is there anything else (not listed in the last question) that you would like to learn?** __________

14. **How much information should patients receive about chronic kidney disease?** (Basic necessary, Basic necessary PLUS some more, A lot of information, Everything that a doctor knows)
15.
16. **You can pick more than one option for this question. I learn the best by using:** (Lectures, Classes with other patients with kidney disease, A smartphone app, Videos, Computer, Pamphlets and brochures)
17. **Your kidney doctor's team plans on organizing classes to teach patients about chronic kidney disease. Would you have interest in attending these classes?** (Yes, No)
18. **I would not want to attend classes because:** (My work schedule may conflict with these sessions, I can't arrange for transportation to attend classes, I know enough information about chronic kidney disease and won't learn much more from these classes, Lack of time, Cost if not covered by insurance, Family commitments, Other reasons: __________)
19. **What barriers do you anticipate for yourself or other patients in attending such a program? __________**
20. **You can pick more than one option for this question. I learn the best by using:** (Lectures, Classes with other patients with kidney disease, A smartphone app, Videos, Computer, Pamphlets and brochures)
21. **If we decide to do a group education class, what is the MINIMUM number of sessions you are willing to attend?** (1, 2, 3, 4, 5, 6)
22. **If we decide to do a group education class, what is the MAXIMUM number of sessions you are willing to attend?** (1, 2, 3, 4, 5, 6)
23. **If we decide to do a group education class, how long should the class be?** (1 hour, 2 hours, 3 hours, Other: _____)
24. **Would you prefer your caregivers and/or family members attend these classes with you?** (Yes, No)
25. **Who should be teaching the education class?** (A kidney doctor, A trained nurse, A trained patient with kidney disease, Other: _______)
26. **Should there be an option to connect with other patients with kidney disease to create a peer support system?** (Yes, No)
